# Utilisation of mental health services before, during, and after COVID-19 restrictions: interrupted time-series analysis in England

**DOI:** 10.1186/s12913-026-14362-z

**Published:** 2026-04-01

**Authors:** Campbell Robertson, Julii Brainard, Gillian E. Smith, Sally E. Harcourt, Uy Hoang, Alex J. Elliot, Simon de Lusignan, Felipe J. Colón-González, Iain R. Lake

**Affiliations:** 1 National Institute for Health and Care Research Health Protection Research Unit in Emergency Preparedness and Response, King’s College London, London, UK; 2 School of Environmental Science, University of East Anglia, Norwich, UK; 3 Department of Psychiatry, University of Oxford, Oxford, UK; 4 Norwich Medical School, University of East Anglia, Norwich, UK; 5 Real-Time Syndromic Surveillance Team, Field Services, Chief Medical Advisor Group, UK Health Security Agency, Birmingham, UK; 6 Nuffield Department of Primary Care Health Sciences, University of Oxford, Oxford, UK; 7 Royal College of General Practitioners, London, UK; 8 Data for Science and Health, Wellcome Trust, London, UK

**Keywords:** Pandemic preparedness, Depression, Anxiety, Mental health, COVID-19, Primary care, Utilisation, Psychiatric epidemiology, Syndromic surveillance, ITSA

## Abstract

**Background:**

During the Coronavirus Disease 2019 (COVID-19) pandemic, the World Health Organisation reported a 25% increase in anxiety and depression, and multiple studies indicated that COVID-19 experiences might increase the prevalence of mental illness with subsequent high demands on mental health (MH) services. However, few studies have focussed upon MH across the entire period of pandemic restrictions within England or considered implications for pandemic preparedness.

**Methods:**

We conducted an interrupted time-series analysis of MH service utilisation across England’s National Health Service, including primary care consultations, emergency department attendances, and telephone advice line contacts. The study period was January 1st 2019 to April 20th 2022. Using data from before and after pandemic restrictions, negative binomial regression models generated expected MH utilisation if the pandemic had not occurred. Expected and observed MH utilisation were compared. MH service indicators were analysed both overall and stratified by age group.

**Results:**

Early restrictions saw significant declines in access to MH services, telephone calls for MH advice reduced by 36.8% (95% CI -42.0, -31.9) and general practitioner (GP) in-hours consultations for depression decreased by 64.6% (95% CI -77.8, -53.3). Later restrictions revealed an increase in consultations in primary care for anxiety, with an increase of 41.8% (95% CI 38.7, 44.7) in GP out-of-hours. By the final period of restrictions, most MH indicators had either returned to expected levels or were significantly above expected presentations. Young people (15–24) exhibited MH utilisation differences —sharply reduced anxiety and MH during initial restrictions but increasing anxiety in later restrictions within primary care.

**Conclusions:**

COVID-19 restrictions were associated with overall decreases in the utilisation of MH services but increases from in person to remote services were observed. For future pandemic preparedness, remotely accessible MH services are important when in-person services are reduced and the surveillance sources used in this study offers the possibility of real-time decision making.

**Trial registration:**

The data used in this study are based on patients accessing healthcare services in England and are therefore retrospectively registered.

**Supplementary Information:**

The online version contains supplementary material available at 10.1186/s12913-026-14362-z.

## Introduction

Mental health (MH) disorders are a leading cause of global disease [1–3]. During the Coronavirus Disease 2019 (COVID-19) pandemic, the World Health Organization (WHO) reported a 25% increase in anxiety and depression [4], which was reinforced by multiple studies predicting that COVID-19 might lead to increases in prevalence or severity of mental illness [5–7]. However, most literature on MH morbidity published during COVID-19 was based on self-reported surveys, which are prone to subjective bias and only investigated outcomes at a single time point. Given the dynamic nature of COVID-19, it is crucial to understand how the pandemic affected MH services using less subjective data, that is collected consistently over a long period.

Expectations that COVID-19 would be detrimental to MH were common and reasonable [5–7]. Non-pharmaceutical interventions such as lockdown measures and social distancing disrupted everyday life, potentially negatively affecting MH [8]. Social isolation and physical quarantine were linked to damaging psychological impacts [9]. For many there was uncertainty about how much COVID-19 mortality or morbidity would personally affect them, while daily news reports kept people aware of the disturbing facts of rising COVID-19 cases, deaths and indicators of economic recession [10–13].

However, findings about the impact of COVID-19 on MH are inconsistent. Early cross-sectional studies suggested high mental illness burdens. Xiong et al. [13] conducted a systematic review of 19 cross-sectional studies from 8 countries on the impact of COVID-19, finding that COVID-19 was associated with high levels of psychological distress. In a systematic review and meta-analysis of UK studies, Dettmann et al. [14] found that the prevalence of anxiety during the first lockdown (March-May 2020) was 31% (95% CI 26%-35%) compared with a prevalence of 4.65% pre-pandemic. That study also found that prevalence of depression was high at 32% (95% CI 29–35%) compared to 4.12% pre-pandemic. However, later systematic reviews reported mixed impacts on the prevalence of MH conditions during COVID-19 [15–16].

Limitations and possible biases affect many MH studies undertaken during the COVID-19 pandemic. Inherent to cross-sectional design is a single time point. Even some longitudinal studies may have only looked at MH a few times during the pandemic missing the dynamic nature of COVID-19. Cross-sectional and many longitudinal studies are typically reliant on samples of convenience and can suffer from selection bias. Hence, using the most convenient recruitment methods, there is high likelihood of oversampling of persons with a health condition, leading to over-estimates of incidence and/or prevalence.

Ongoing patient utilisation for MH services is a novel method that can be used to indicate mental illness morbidity and provide trends over time. Specific to COVID-19, Smith et al. [17] produced an observational study of daily MH presentations across multiple healthcare settings during the first 9 months of COVID-19 in England. They found a significant decrease in MH presentations across four types of healthcare services from March to September 2020. Carr et al. [18] used primary care data from the UK Clinical Practice Research Datalink (CPRD) to estimate MH morbidity from January to September 2020. They found that first-presentation incidence of common MH conditions and prescriptions were 36% to 48% lower in April 2020 but had returned to expected incidence by September 2020. Mansfield et al. [19] also used patient records from the CPRD to monitor weekly contact rates for MH conditions between 2017 and 2020. Contact rates for multiple conditions stayed low and had not returned to expected levels by July 2020. These decreases in utilisation for MH services were noted in other primary care studies within England through to autumn 2020 [20, 21]. However, none of these studies looked across the whole period of COVID-19 restrictions and few looked at multiple sources of patient utilisation data for MH services.

Here we create a comprehensive overview of the impact of COVID-19 restrictions upon multiple MH services in the National Health Service (NHS) in England. Daily data from four different services through which the public may access MH services were acquired. Data were obtained for a long period pre and post pandemic restrictions. We analysed several different MH presentations subdivided by age group. Additionally, the utility of MH service data for event analysis and future pandemic preparedness is considered.

## Methods

We applied an interrupted time series design to explore changes in MH utilisation during COVID-19 restrictions using UK Health Security Agency (UKHSA) national syndromic surveillance systems (SSS) previously described [17] and electronic healthcare records (EHRs). Daily data were obtained January 1st 2019 to April 20th 2022. Analysis was stratified by 4 specific health services.

### Data sources and MH conditions

Data on MH service utilisation were accessed through SSS routinely monitored by UKHSA. These were anonymised surveillance data for: NHS healthcare advice triaged telephone calls (NHS 111); general practitioner (GP) out-of-hours (GPOOH) consultations; and emergency department attendances (EDSSS). Each dataset comprised requests for care or advice rather than describing treatment or treatment outcomes. We also collected the total utilisation of these systems to explore any change in the capacity (supply). Analysis from another UKHSA SSS, the National Ambulance Syndromic Surveillance System (NASSS) can also be viewed in the supplementary material (Table S5 & Figure S2) but is not included in the main text due to limited MH activity.

EHRs for GP consultations during ‘in-hours’ (working days and hours) services (GPIH) were available from Oxford-Royal College of General Practitioners (RCGP) Clinical Informatics Digital Hub (ORCHID), a trusted research environment that holds the Oxford-RCGP Research and Surveillance Centre (RSC) sentinel network [22]. GPIH data are an extract of EHR data from a sentinel network of over 2,000 primary care practices in England [23, 24]. The GPIH data describe activity for patients who were registered as of January 1st 2019 and the prescription data exclude newly registered patients or new start medication courses started after this date. GPIH data are recorded in SNOMED clinical terms and a list of codes used for this study is included in supplementary files. Total consultations for the GPIH system were unavailable.

From these four datasets, MH indicators were extracted (Table 1; details in Tables S1-S3). In this main article, we refer to presentations related to general MH and common MH conditions (Anxiety and Depression). Analysis for other MH conditions and indicators (Prescriptions, Self-harm, Sleep Difficulties, Alcohol Intoxication and Overdoses) can be viewed in the supplementary material (Table S4). For simplicity, we refer to presentations, calls, attendances, and consultations collectively as ‘indicators’.

**Table 1:** Indicators for mental health category indicator counts in respective services

System	Indicator	Mean Daily Indicators	Stratified by Age (Y/*N*)
NHS 111	Total Calls Mental Health Problems	43,022 539	N Y
GPOOH	Total Consultations All Mental Health	25,900 118	N Y
	Anxiety	64	N
	Depression	20	N
EDSSS	Total Attendances All Mental Health	22,385 441	N Y
GPIH	Total Consultations All Mental Health	N/A 622	N/A Y
	Anxiety	193	Y
	Depression	137	Y

Notes: NHS 111 – National Health Service 111 telephone service; GPOOH – General Practitioner Out-of-Hours; EDSSS – Emergency Department Syndromic Surveillance System; Surveillance; GPIH – General Practitioner In-Hours; N/A – Not Available

### Sub group stratification

Subgroup analyses for age group were done for most common conditions (Table 1), as long as they also had ≥ 100 average daily indicators. Total indicators were not stratified by age to prioritise the analysis of MH indicators across age groups. Analysis by sex can be seen in the supplementary material (Figure S4 and S5). Age sub-groups were: 15–24, 25–44, 45–64, 65–74, 75 + years. There were low counts of persons under age 15 in these datasets and therefore these groups were excluded.

### Time periods

Monitoring dates were chosen using pragmatic and objective criteria. Data from before and after pandemic restrictions were required from a long but relatively recent period that captured seasonal, day of week and holiday effects on ‘normal’ service utilisation. The pre-pandemic restrictions period (PRE) was defined as January 1st 2019 to February 24th 2020, to capture a full year of ‘normal’ utilisation. Our post pandemic restrictions period (POST) was from the lifting of all restrictions in England (July 17th 2021) until the end of our period of study (April 20th 2022). We acknowledge that the WHO did not declare the end of the COVID-19 pandemic until May 2023. However, we wanted to analyse the impact of social restrictions in England on MH service utilisation which ended much sooner than the WHO announcement.

Between the PRE and POST periods we defined five COVID-19 restriction periods varying by degrees of social restrictions. PRL1 (Pre-Lockdown Period 1), spanned 25th February 2020 to 22nd March 2020, before social distancing was legally enforced. During PRL1, healthcare services were required to balance infection control with access for patients, and GPs were advised to limit in person contact [18]. Furthermore, public awareness of COVID-19 rose sharply during PRL1 due to news reports and new social-distancing and self-isolation guidelines [25–27]. This heightened awareness likely influenced healthcare-seeking decisions.

Four later periods were identified using a timeline of UK government coronavirus lockdowns and government guidance [28]: L1 (lockdown 1, 23rd March 2020 to 31st of May 2020), PL1 (post-lockdown 1, 1st June 2020 to 4th November 2020), L2 (lockdown 2, 5th November 2020 to 7th March 2021), PL2 (post-lockdown 2, 8th March 2021–18th July 2021). L2 incorporates a November 2020 four-week-duration lockdown alongside a lockdown starting in January 2021. We acknowledge that healthcare-seeking patterns may not follow these specific dates exactly. These periods are simplifications to enable national analysis, and do not capture varying localised social contact and self-isolation regulations. Both lockdown periods in this study represent stricter social restrictions, while the post lockdown periods represent relaxed restrictions.

### Analysis

Key to our analysis was the generation of counterfactual estimates for MH indicators – which make a prediction if the COVID-19 pandemic/restrictions had not occurred, using data from PRE and POST pandemic restriction periods. This was chosen as our control method, as it is considered the most appropriate control for an interrupted time-series design [29]. These estimates were compared to observed presentations for the 5 COVID-19 restriction periods (PRL1, L1, PL1, L2, PL2).

To model counterfactual estimates, negative binomial regression models were adopted due to over-dispersion of indicator counts. Models were fitted in R using the ‘MASS’ package [30]. Long-term linear trends were controlled for by including a sequential date indicator variable (1 to 1206). Day-of-the-week (DOW) effects were accounted for using a categorical variable (1–7). Public holidays (Bank) were controlled using a Boolean variable. Seasonal trends were modelled with a categorical variable representing each calendar month (1–12). Only indicator counts from the PRE and POST periods were included as dependent variables in the models to generate counterfactual estimates.

The counterfactual model equation is:

$$\begin{aligned}\mathrm{log}\left(\mu\right)&=a+\sum\nolimits_{}^{5}{\beta}_{i}*{x}_{i}+\sum\nolimits_{k=1}^{K}f\left({X}_{k}\right)\cr & \quad+DOW+Month+Bank\end{aligned}$$

Where µ is the natural logarithm of the mean MH indicator (dependent variable); α represents the intercept and a set of 5 linear variables ${x}_{i}$ each associated with their own coefficients ${\beta}_{i}$; i = 1:5 representing the 5 different linear variables; X is a matrix of K = 2 piecewise linear functions representing the segmented (PRE & POST) linear trend, defined as spline functions, f(X_k_). We evaluated various time-trend transformations (linear, quadratic, and splines) using AIC and deviance, finding similar performance but selecting a linear term for its simplicity and best visual fit [31].

Our main analysis addresses general MH indicators, and the two most common MH conditions: anxiety and depression. Supplementary files document the less common MH-related indicators (self-harm, overdoses, alcohol intoxication, sleep difficulties and prescriptions for MH medications). Data do not indicate if the overdose, self-harm or alcohol intoxication were deliberate or accidental. Analysis stratified by sex (Male & Female) can also be viewed in the supplementary material. Differences between actual and expected indicator counts were calculated alongside percentage change from expected values. Results are reported for all population, as well as age group. Confidence Intervals (95% CI) for percentage change were calculated by applying the “qnorm” function in R. We interpret 95% confidence intervals for the percentage difference between actual and counterfactual as significant when they are entirely above or below zero. All analysis was undertaken in R Version 4.3.0.

## Results

**Fig. 1:**
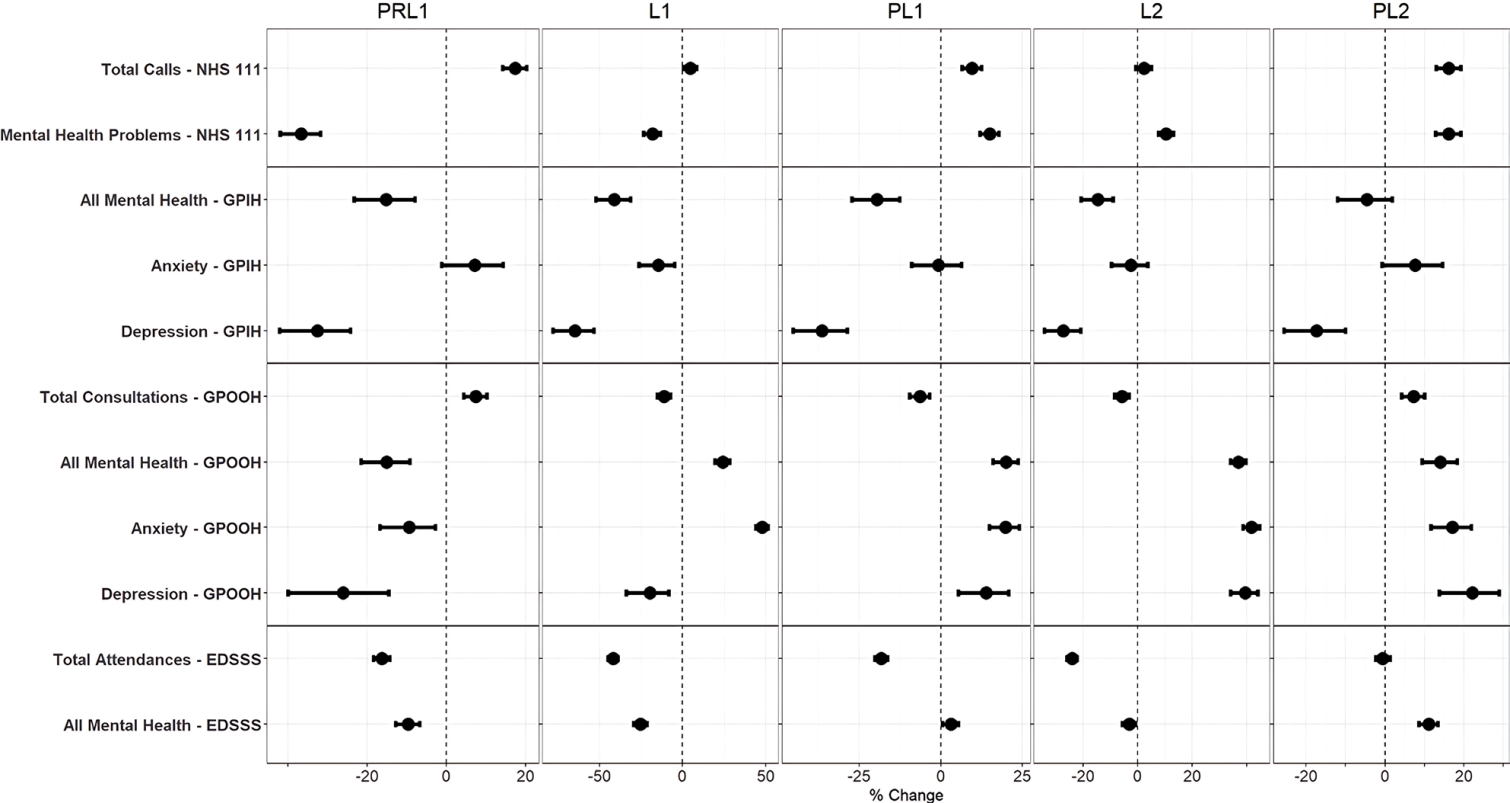
Forest plot of selected mental health and total indicators by syndromic surveillance system. Notes: Percentage change comparing actual counts with counterfactual estimates is presented on the x-axis, with a dotted vertical line during each period representing 0% change. PRL1: Pre-lockdown 1: February 25 to March 22, 2020; L1: Lockdown 1: March 23 to May 31, 2020; PL1: Post Lockdown 1: June 1 to November 4, 2020; L2: Lockdown 2: November 5, 2020 to March 7, 2021; PL2: Post-Lockdown 2: March 8 to July 7, 2021. NHS 111 – National Health Service 111 telephone service; GPIH – General Practitioner In-Hours; GPOOH; General Practitioner Out-of-Hours; EDSSS – Emergency Department Syndromic Surveillance System

**Fig. 2:**
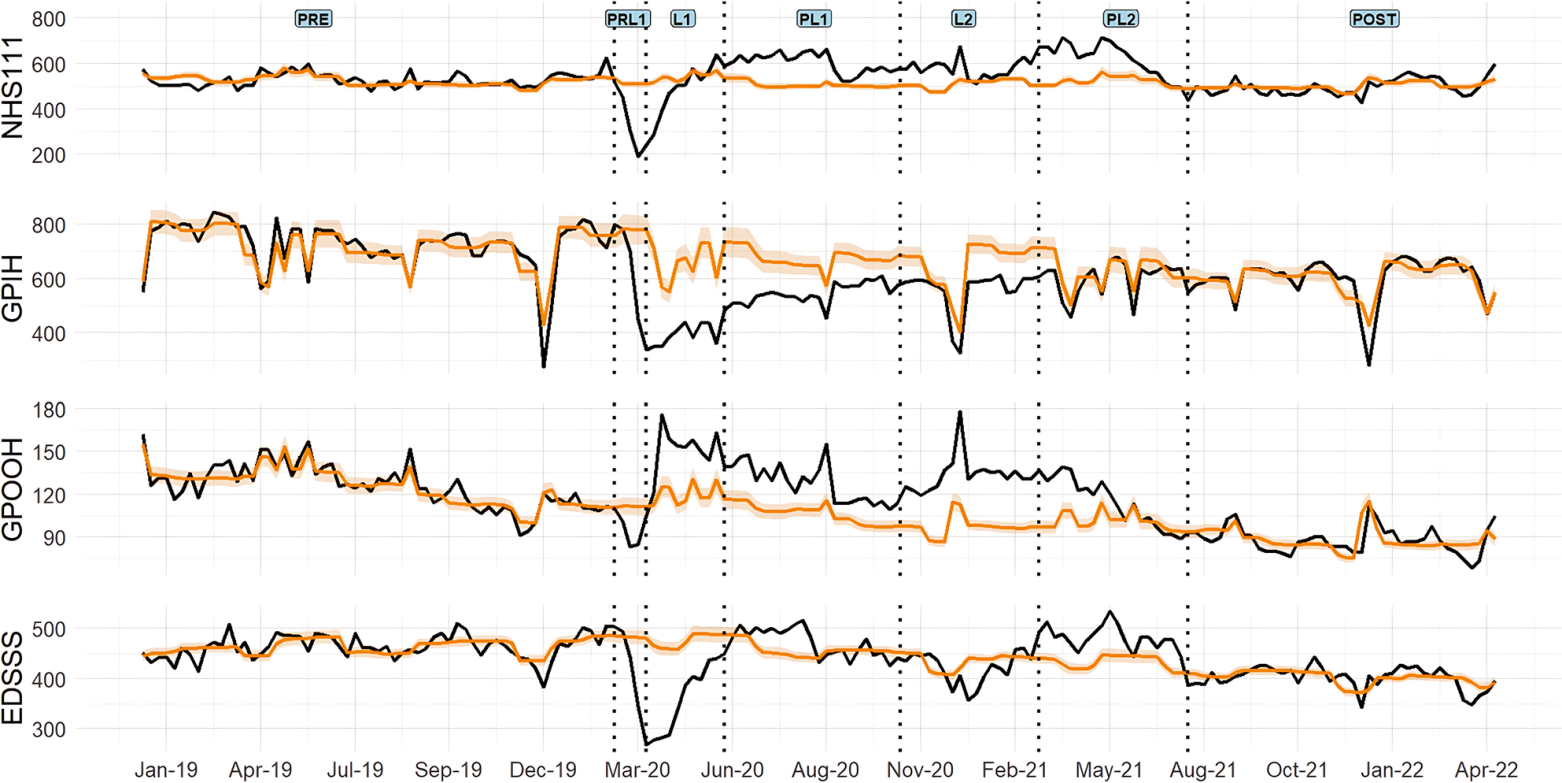
Indicators for all mental health conditions. Notes: Black solid line represents actual indicator counts, orange line represents the modelled (counterfactual) indicator counts with shading for 95% CI. The indicator counts are daily totals. Vertical lines indicate period boundaries. Counterfactual estimates and actual indicator counts are shown for ‘All mental health conditions’ from four of the NHS services. NHS 111 – National Health Service 111 telephone service; GPIH – General Practitioner In-Hours; GPOOH; General Practitioner Out-of-Hours; EDSSS – Emergency Department Syndromic Surveillance System

Differences between actual and counterfactual counts in each of the five COVID-19 periods of restrictions were assessed quantitatively (Table 2) and selected MH indicators were visualised using a forest plot (Fig. 1) - total indicators are also included to compare how supply of each system compared with MH indicators. Quantitative analysis of other MH indicators can be viewed in Table S5. Figure 1 shows that MH problems for the NHS 111 system showed a significant decrease during the PRL1 period (-36.8%), compared with the totals for this system which increased substantially (17.3%). Calls for MH problems were elevated in later pandemic periods (PL1, L2, PL2) – however, were only significantly higher than total calls during the L2 period (10.5%). A time-series for calls to NHS 111 for MH problems can be seen in Fig. 2.

**Fig. 3:**
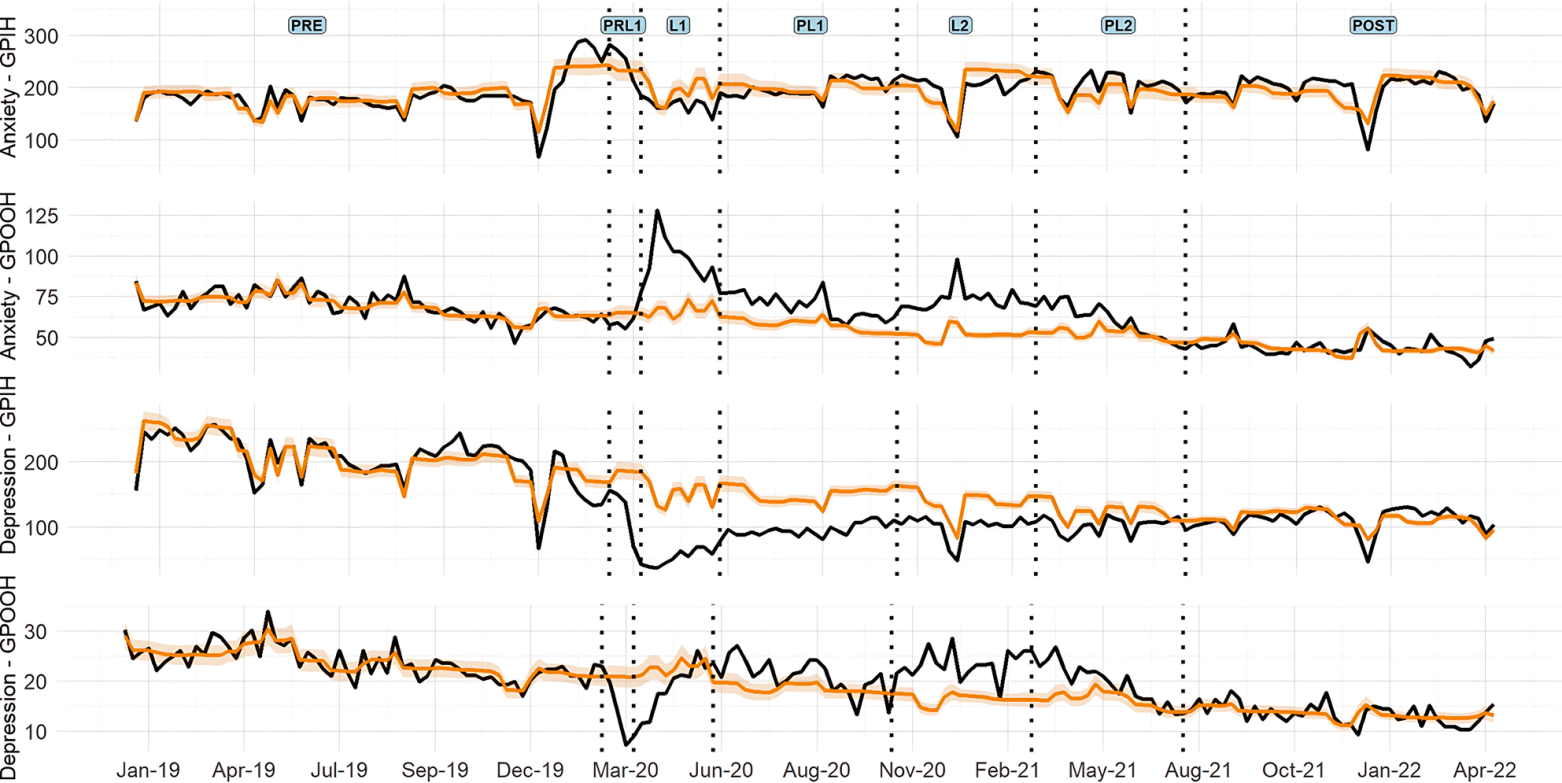
Anxiety and depression indicators. Notes: The black solid line represents actual indicator counts, and the orange line represents modelled (counterfactual) indicators, with shading showing the 95% CI. The indicator counts are daily totals. Vertical lines indicate monitoring period boundaries. Comparable all-population data for anxiety and depression indicators are from the GPIH and GPOOH. GPIH – General Practitioner In-Hours; GPOOH – General Practitioner Out-of-Hours

GPIH consultations for anxiety remained at counterfactual levels across all periods of restrictions, except L1. While the other indicators witnessed a large decrease in consultations during the PRL1, L1 and PL1 periods – with consultations for depression showing the largest decrease at 64.6%. All MH then returned to counterfactual estimates during the L2 period. While consultations for depression remained below expected estimates in all periods of restrictions. A time-series for All MH consultations to the GPIH can be seen in Fig. 2 – while a time-series for anxiety and depression can be seen in Fig. 3.

MH indicators for the GPOOH system also decreased during the PRL1 period, while the totals for this system increased (7.4%). Following this period, All MH and anxiety were higher than counterfactual expectations and total consultations in subsequent periods – with anxiety showing the highest increase of 41.8% during L2. Consultations for depression remain below counterfactual levels during the L1 period - before also increasing compared to counterfactual and total presentations in the PL1 period. A time-series for All MH consultations to GPOOH can be seen in Fig. 2 – while a time-series for anxiety and depression can be seen in Fig. 3.

Within the EDSSS system, the All MH indicator and the total indicator were below counterfactual estimates during PRL1 and lowest in the L1 period (25.4%). During the PL1 period, MH estimates had returned to counterfactual estimates and were above total indicators. The L2 period witnessed a decrease in MH while the PL2 period shows MH indicators were increased (11.1%) when compared with counterfactual and total presentations. A time-series for All MH attendances to the EDSSS can be seen in Fig. 2.

Time series for NASSS ambulance callouts related to overdoses, EDSSS attendances for overdoses and alcohol intoxication, GPOOH consultations for self-harm, NHS 111 calls for sleep difficulties, and GPIH prescriptions were visualised (Figures S1-S2). All indicators showed trends of long-term falls except for sleep difficulties which had an upward trend. However, the mean number of calls for sleep difficulties was low (*n* = 32).

**Table 2:** Percentage differences between actual and counterfactual utilisation for all-population indicators

**System**	**Period \ Indicator**	**PRL1**	**L1**	**PL1**	**L2**	**PL2**
EDSSS	Total Attendances	-16.3 (-18.5, -14.3)	-41.8 (-44.7, -39.0)	-18.2 (-20.1, -16.3)	-24.1 (-25.9, -22.3)	-0.6 (-2.4, 1.2)
	All Mental Health	-9.7 (-12.9, -6.8)	-25.4 (-29.5, -21.7)	3.1 (0.6, 5.4)	-3.1 (-5.5, -0.9)	*11.1 (8.5, 13.4)*
GPIH	All Mental Health	-15.2 (-23.4, -8.0)	-40.9 (-52.1, -31.2)	-19.5 (-27.2, -12.7)	-14.6 (-20.8, -9.0)	-4.6 (-12.0, 1.8)
	Anxiety	7.2 (-1.2, 14.3)	-14.5 (-26.3, -4.8)	-0.8 (-9.0, 6.3)	-2.5 (-9.5, 3.7)	7.6 (-0.8, 14.6)
	Depression	-32.7 (-42.2, -24.3)	-64.6 (-77.8, -53.3)	-36.4 (-45.2, -28.7)	-27.2 (-34.2, -21.0)	-17.3 (-25.6, -10.1)
NHS111	Total Calls	17.3 (14.2, 20.3)	4.5 (0.4, 8.3)	9.6 (6.5, 12.5)	2.3 (-0.7, 5.1)	16.2 (13.0, 19.2)
	Mental Health Problems	-36.8 (-42.0, -31.9)	-18.1 (-23.3, -13.4)	15.0 (12.0, 17.7)	10.5 (7.8, 13.1)	16.1 (12.8, 19.2)
GPOOH	Total Consultations	7.4 (4.4, 10.2)	-11.3 (-15.4, -7.5)	-6.4 (-9.5, -3.5)	-5.8 (-8.5, -3.2)	7.2 (4.2, 10.0)
	All Mental Health	-15.1 (-21.6, -9.3)	24.1 (19.4, 28.3)	20.0 (16.0, 23.7)	37.0 (34.2, 39.6)	14.0 (9.3, 18.2)
	Anxiety	-9.4 (-16.8, -2.8)	47.8 (43.9, 51.2)	19.8 (14.9, 24)	41.8 (38.7, 44.7)	17.1 (11.6, 21.9)
	Depression	-26.1 (-40.1, -14.6)	-19.7 (-33.9, -8.2)	13.8 (5.4, 20.8)	39.5(34.2, 44.1)	22.1 (13.8, 28.9)

**Notes**: Values are % difference with 95% confidence intervals. **PRL1**: Pre-lockdown 1: February 25 to March 22, 2020; **L1**: Lockdown 1: March 23 to May 31, 2020; **PL1**: Post Lockdown 1: June 1 to November 4, 2020; **L2**: Lockdown 2: November 5, 2020 to March 7, 2021; **PL2**: Post-Lockdown 2: March 8 to July 7, 2021. NHS111 – National Health Service 111 telephone service; GPIH – General Practitioner In-Hours; GPOOH; General Practitioner Out-of-Hours; EDSSS – Emergency Department Syndromic Surveillance System

**Fig. 4:**
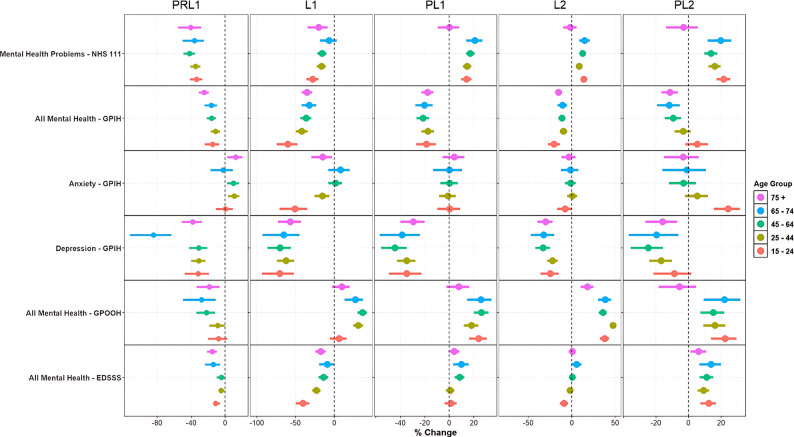
Forest plot of mental health indicators stratified by age group. Notes: Percentage change comparing actual counts with counterfactual estimates is presented on the x-axis, with a dotted vertical line during each period representing 0% change. PRL1: Pre-lockdown 1: February 25 to March 22, 2020; L1: Lockdown 1: March 23 to May 31, 2020; PL1: Post Lockdown 1: June 1 to November 4, 2020; L2: Lockdown 2: November 5, 2020 to March 7, 2021; PL2: Post-Lockdown 2: March 8 to July 7, 2021. NHS 111 – National Health Service 111 telephone service; GPIH – General Practitioner In-Hours; GPOOH; General Practitioner Out-of-Hours; EDSSS – Emergency Department Syndromic Surveillance System

Figure 4 shows MH indicators stratified by age group. This analysis was only undertaken when there were sufficient numbers in each age category (Table 1). In most periods, there are few apparent differences for age subgroups. The most striking age-related differences were for persons aged 15–24 years compared to other ages. Persons aged 15–24 years in L1 had especially low ED attendances for all MH conditions and low GPIH attendances for anxiety during L1, but especially high GPIH consultations for anxiety during PL2. Consultations for depression are also substantially reduced in the 65–74 group during the PRL1 period.

Forest plots for other MH indicators subdivided by age group can be viewed in the supplementary material, Figure S3. MH indicators subdivided by sex can be viewed in the supplementary material, Figure S4 and S5. There is evidence of increased anxiety consultations among females in the GPIH system during the study period, although these differences are not statistically significantly different from male consultations.

## Discussion

We show an overall decline in utilisation of MH services during COVID-19 restrictions within England. As COVID-19 restrictions progressed there was a shift in utilisation from in-person services (GPIH, EDSSS) to remote services (NHS 111, GPOOH). The final period of restrictions (PL2) demonstrates that MH indicators for NHS 111, GPOOH, and EDSSS are all elevated above expected levels – which may be due to delayed seeking MH support from earlier periods. However, total indicators for both NHS 111 and GPOOH are not significantly different from MH indicators, which may indicate delayed healthcare seeking behaviour in general instead of a specific impact on MH. Shifts from in-person services to remote services, highlight the importance of MH services having complementary in-person and remote access points during health crises. Increases in COVID-19-related and acute respiratory presentations during the pandemic were associated with substantial pressures on service capacity and care pathways [19]. Such system-level disruptions may have contributed to reductions in non-COVID presentations, including MH attendances particularly during the early phases of the pandemic.

Considering overall GPIH saw a reduction during restrictions, consultations for anxiety were relatively stable during most periods of restrictions and were increased in the GPOOH service. This may indicate increased anxiety prevalence in the general population during COVID-19 restrictions, as seen in primary care consultations – a systematic review involving multiple countries found increased social anxiety during the COVID-19 pandemic [32]. Conversely, primary care consultations for depression witnessed large reductions during all periods of restrictions and it is unlikely the increase in consultations within GPOOH offset this reduction. This indicates unmet demand for individuals seeking consultations for depression within primary care – untreated depression for long periods of time is associated with exacerbation of symptoms as well as less effective outcomes when treatment is received [33].

There was evidence for age-specific differences in utilisation of MH services in this study. Notably there appears to be a large reduction in the 15–24 group within primary care for all MH and anxiety during the first lockdown. The final period of restrictions (PL2) showed primary care consultations for anxiety were substantially higher in the 15–24 age group, this effect is also seen to a lesser extent with all MH. The reasons for this effect in the youngest age group is unclear but may be that this age group was disproportionately impacted by service changes within primary care earlier in the pandemic, which resulted in a surge in seeking MH support during the final phase of restrictions. It may also indicate the culminative effect of social restrictions or a rise in social anxiety as normal interactions and responsibilities resume. Results from an Australian cohort study found that prevalence of psychological distress during the COVID-19 pandemic was highest amongst young people, particularly females. Some explanations for this may be due to unemployment, financial insecurity, education disruption, lack of social support, and physical and social isolation as factors that disproportionately impacted younger people [34].

A decrease in utilisation of MH services within the UK during early COVID-19 restrictions has been documented elsewhere [17–21, 35, 36]. However, the increase in MH presentations to remote services during later restrictions, alongside the increase in MH and anxiety presentations in the young were not previously documented. In contrast to our research, few other studies used datasets from multiple healthcare services to observe changes in utilisation during COVID-19 restrictions and many studies have not explored impacts over the entire period of restrictions.

Silva-Valencia et al. [37] conducted an interrupted time-series analysis of MH presentations to primary care across nine countries (excluding UK). Their study found increased demand for MH services, contrasting with our findings of decreases within primary care during COVID-19. Silva-Valencia et al. [37] employed monthly MH visit rates compared to total visits as their primary outcome. This allowed international comparisons, but potentially inflated rates if total primary care consultations reduced at a higher rate. This can be seen in our analysis for the EDSSS – where MH attendances are reduced but are still higher than total attendances. Alternatively, our use of absolute counts rather than visit-rate ratios may have underestimated MH service demand, as we analysed raw presentation numbers rather than their proportion to total visits - though we did account for overall attendance trends by including total presentations across most services where possible.

Future studies should investigate the impact of the MH service reductions documented in this study. While we focused on the immediate impact of social restrictions, it is likely that COVID-19 continued to affect services after restrictions lifted in England, or that individuals experienced a delayed MH response – a recognised phenomenon following potential trauma or crises [38]. The Adult Psychiatric Morbidity Survey (APMS), the gold standard for MH prevalence data in England, shows a disproportionate rise in common MH conditions among 16–24 year olds, increasing from 18.9% in 2014 to 25.8% in 2023/4 [39]. This increase is likely due to a multitude of factors beyond the scope of this research – however, our findings of disproportionate primary care reductions and subsequent increases in this age group suggest that future research is essential to determine the specific impact of service availability on MH prevalence rates. Additionally, research should employ intersectional analysis, looking at the interplay of age group and sex, as well as important social demographic and determinant factors.

The main strength of this study is the use of diverse healthcare settings to represent how the general population utilised MH services within England – with the GPIH primary care consisting of nearly 10 million patients and the NHS 111 system being available at a national level. These datasets allowed us to witness shifts in how people were utilising MH services during COVID-19 restrictions. Analysis also benefited from high consistency in data collection methods over the period, enabling us to capture trends from before and after COVID-19 restrictions. A further strength is that the majority of MH indicators employed were derived from clinical need, reflecting MH outcomes following formal consultations. However, it is important to note that particularly NHS 111 data reflects advice-seeking behaviour rather than formal clinical consultations.

There are a number of limitations associated with this study. As mentioned previously, we only analyse utilisation during periods of COVID-19 restrictions and do not analyse after these restrictions were lifted. Furthermore, although this study included a number of different healthcare settings, individuals may have accessed care through alternative settings, including digital MH tools or third-sector services, and the datasets used will not capture those who avoided or delayed seeking support after the study period. We noted inconsistencies in coding for some systems, such as GPOOH, only 38% of presentations contained a diagnosis code, meaning a considerable number of MH consultations may have been lost in this system due to coding error. Carr and colleagues note that some of the reduction in primary care may be a result of inaccuracies in coding that GPs had to make to adapt to remote consultation methods [18] – therefore changes witnessed in this study may reflect recording artefacts as opposed to true changes in utilisation. Syndromic surveillance data lack detailed information on symptom severity, duration, diagnostic specificity and cannot distinguish between mild and severe MH presentations. There were additional factors that this study was not able to analyse such as differential effects with preexisting mental illness, ethnicity, socio-economic status, and employment status. It is likely that certain sub-groups were disproportionately impacted by COVID-19 restrictions and we were not able to explore these as syndrome based data does not include details of these groups. Maddock et al. [40] revealed several disadvantaged groups within the UK that experienced a higher rate of healthcare disruption during pandemic restrictions. To ensure consistent data availability for analysis across the entire study period, we included only suppliers who supplied daily data continuously from the start date (1st January 2019). However, this criterion excluded suppliers who began supplying data after the start date, and consequently, any patients who accessed services solely through those new providers during the study period. Additionally, the methods used in this study are observational and attempts to establish causality should be interpreted with caution.

These results demonstrate how syndromic surveillance, combined with real-world data sources like EHRs, can effectively monitor community MH service utilisation - particularly during nationwide crises. For future pandemic preparedness, such systems offer real-time decision-making capabilities and can reveal demographic-specific utilisation patterns. However, as established in this study and other studies on syndromic surveillance [17, 41] – one of the challenges is understanding true changes in MH utilisation from general changes in healthcare seeking behaviour.

## Conclusions

This study documents an overall decline in MH service use across England during COVID-19 restrictions, with a significant shift from in-person to remote services. While primary care consultations for depression fell sharply, anxiety presentations increased. Young people (15–24) were disproportionately impacted in primary care, showing initial steep declines followed by later surges in anxiety consultations, suggesting delayed help-seeking or heightened vulnerability. These findings necessitate MH services maintaining both in-person and remote access during crises. We demonstrate syndromic surveillance’s value for real-time MH service monitoring and resource planning in future incidents. Future research must assess the long-term impact of these service disruptions, particularly in relation to a long-term rise in MH prevalence.

## Supplementary Information

Below is the link to the electronic supplementary material.

Supplementary Material 1

## Data Availability

Applications for requests to access UKHSA-held anonymised data should be submitted to (https://www.gov.uk/government/publications/accessing-ukhsa-protected-data). Requests for access to the Oxford-Royal College of General Practitioners Clinical Informatics Digital Hub (ORCHID) sentinel network data can be made through the Primary Care Hosted Research Datasets Independent Scientific Committee: http://www.phc.ox.ac.uk/intranet/better-workplace-groups-committees-open-meetings/primdisc-committee-1/primdisc-committee.

## Abbreviations

COVID-19: Coronavirus disease 2019
ED: Emergency department
EDSSS: Emergency department syndromic surveillance system
HER: Electronic Healthcare Record
GP: General practice (primary care providers)
GPIH: General practice in-hours (service)
GPOOH: General practice out-of-hours (service)
L1: Lockdown 1 period (March 23 to May 31, 2020)
L2: Lockdown 2 period (November 5, 2020 to March 7, 2021)
MH: Mental health
NASSS: National Ambulance Syndromic Surveillance System
NHS: National Health Service
NHS 111: National Health Service telephone advice service
PL1: Post Lockdown 1 period (June 1 to November 4, 2020)
PL2: Post-Lockdown 2 period (March 8 to July 7, 2021)
POST: Post-pandemic (July 19, 2021 to April 20, 2022)
PRE: Pre-pandemic (January 1, 2019 to February 24, 2020)
PRL1: Pre-lockdown 1 period (February 25 to March 22, 2020)
RSC: Oxford-RCGP Research and Surveillance Centre
SNOMED: Systematized Nomenclature of Medicine
SSS: Syndromic surveillance system
UKHSA: United Kingdom Health Security Agency
