# Supplementary material for: Utilisation of mental health services before, during, and after COVID-19 restrictions: interrupted time-series analysis in England
Source: BMC Health Serv Res. 2026 Apr 1;26:457. doi: 10.1186/s12913-026-14362-z (PMC13045032; doi:10.1186/s12913-026-14362-z)

**Table S1:** Description of the syndromic surveillance systems used in the study, all in England UK

| **System** | **Coverage** | **Description** | **Details and Caveats** |
| --- | --- | --- | --- |
| **UKHSA Remote Health Advice Syndromic Surveillance System (NHS 111 calls)** | National (c) | A UKHSA syndromic surveillance system which uses fully anonymised, daily data on calls to NHS 111 (England) | Calls to NHS 111 triaged using NHS Pathways. The syndromic dataset excludes calls to NHS 111 where an immediate threat to life was identified and other calls that have not been triaged using NHS Pathways (this may include calls for repeat prescriptions, information etc). The data are sourced from the NHS 111 Repeat Caller Database |
| **ORCHID primary care dataset of the Royal College of General Practitioners (RCGP) Research and Surveillance Centre (RSC) sentinel network (a)** | Sentinel (c) | A network of general practices, which extracts data from the computerised medical record systems of over 500 practices in England. This network provides a representative sample of the population of England in terms of demographics and clinical outcomes | Data included consultations, diagnoses, symptoms, and prescriptions for common mental health conditions recorded in electronic healthcare records (EHRs) in the network of practices. GP in-hours consultations were based upon a total of 783 practices, which included 9,957,196 registered patients |
| **UKHSA**  **GP Out-of-Hours Syndromic Surveillance System** | Sentinel (c) | Pseudo-anonymised data uploaded from GP out-of-hours service providers who use the Advanced Health and Care Adastra software system and who participate in the UKHSA GP out-of-hours SSS | Only consultation data for 28 out-of-hours service providers that reported consistently throughout the study period were included in the study. Clinical coding varies across the out-of-hours service providers and in this dataset only 39% of consultations had a clinical code |
| **UKHSA National Ambulance Syndromic Surveillance System** | National (c) | Participating ambulance trusts in England reporting daily data to the UKHSA NASSS | Total syndromic calls included all calls where the chief presenting complaint was mapped to one of the syndromic indicators monitored by UKHSA. This does not include all calls submitted to the ambulance trusts. Ten ambulance trusts were included in this study. |
| **UKHSA Emergency Department Syndromic Surveillance System (b)** | Sentinel (c) | UKHSA national EDSSS began operating in April 2018 following introduction of the  Emergency Care Data Set. | 129 Type 1 ED’s which reported daily for the whole of the study period were included. The indicators are based on the primary diagnosis of each attendance. |

Notes:

(a) <https://orchid.phc.ox.ac.uk/> ; <https://bmjopen.bmj.com/content/6/4/e011092>

(b) <https://digital.nhs.uk/data-and-information/data-collections-and-data-sets/data-sets/emergency-care-data-set-ecds>

(c) Difference between national (population) and sentinel surveillance: <https://pmc.ncbi.nlm.nih.gov/articles/PMC3417785/>

**Table S2:** Details of the syndromic surveillance mental health indicators used in this study

| **System** | **Group/ condition** | **Pathway/**  **Read code/**  **SNOMED code** | **Description of indicator** |
| --- | --- | --- | --- |
| **NHS 111 calls** | Mental Health Problems | Pathway | Pathway describing known mental health problems |
|  | Sleep difficulties | Pathway | Sleep difficulties |
| **GP out-of-hours consultations** | Depression | Read code | Depressed; symptoms of depression; low mood; depressive episodes; post viral depression; dysthymia; restlessness and agitation |
|  | Anxiety | Read code | Anxiousness; life crisis; generalised anxiety disorder; acute reaction to stress; panic attacks; Chronic post-traumatic stress disorder |
| **Ambulance call outs** | Overdose/ Ingestion/ Poisoning | NA | Overdoses, ingestion of a substance or poisoning |
| **Emergency department attendances** | Mental health | SNOMED | Dementia; delirium; personality disorder; eating disorder; depressive disorders; anxiety; psychotic disorders; bipolar disorders; schizophrenia; somatization disorder; somatoform disorder; dissociative disorder; adjustment disorder; factitious disorder |
|  | Overdose | SNOMED | Acetaminophen overdose; Non-steroidal anti-inflammatory overdose; overdose of antidepressant drug; sedative overdose; overdose of opiate |
|  | Alcohol intoxication | SNOMED | Alcohol intoxication; alcohol dependence; Uncomplicated alcohol withdrawal; Toxic effect of alcohol; Alcohol withdrawal induced convulsion |

**Table S3.** Details of medication and condition categories, GPIH dataset

| **Indicator** | **Code** | **Medication or condition categories** |
| --- | --- | --- |
| Prescribing | British National Formulary (BNF78, 2019-2020, bnf.org) | AntacidsAndSimeticone ; Benzodiazepine ; MonoamineOxidaseInhibitors  SNRIs ; SSRIs ; TricyclicAntidepressantDrugs |
| Depression | SNOMED CT | Anhedonia Bipolar  AffectiveDisorder  CurrentEpisodeDepression ; FindingOfDepressedMood ; MildMajorDepressionSingleEpisode  MildRecurrentMajor ; DepressionModeratelySevere ; MajorDepressionSingleEpisode  ModerateMajorDepressionSingleEpisode ; ModerateRecurrentMajorDepression  SevereMajorDepressiveDisWithAnxietySingleEpisode ; SevereRecurrentMajorDepression  OrganicMoodDisorder  PostpartumDepression  PsychosisAndSevereDepressionWithAndDueToBPD ; SchizoaffectiveDisorderDepressiveType  SevereRecurrentMajorDepressionWithPsychoticFeature SingleMajorDepressiveEpisodeSevereWithPsychosis  UndifferentiatedSchizophrenia |
| Anxiety | SNOMED CT | FindingOfAnxiety; GeneralisedAnxietyDisorder ; IllnessAnxietyDisorder  ObsessiveCompulsiveDisorder ; OrganicAnxietyDisorder ; PanicDisorder  PosttraumaticStressDisorder |
| All Mental Health Disorders | SNOMED CT | All conditions above for anxiety and depression, *in addition to*  AtypicalDepressiveDisorder  FindingOfDecreaseInAppetite ; FindingOfFatigue ; FindingOfIrritability  FindingOfPsychomotorAgitation  HarmfulUseOfAlcohol ; HarmfulUseOfCannabis ; HarmfulUseOfCocaine ; HarmfulUseOfHallucinogen ; HarmfulUseOfHypnotic ; HarmfulUseOfOpioid ; HarmfulUseOfSyntheticCannabinoid ; SolventMisuse  IntentionallyHarmingSelf ; IntentionalPoisoning ; IntentionalPoisoningByDrug  NightmareDisorder ; NonOrganicDisorderOfTheSleepWakeSchedule ; NonorganicInsomnia  PrimaryHypersomnia  OrganicMoodDisorder  SleepTerrorDisorder ; SleepWalkingDisorder  UndifferentiatedSchizophrenia |

**Table S4.** Indicators for mental health category indicator counts in respective services

| **System** | **Indicator** | **Mean Daily Indicators** | **Stratified**  **by Age (Y/N)** |
| --- | --- | --- | --- |
| **NHS 111** | Sleep Difficulties | 32 | N |
| **GPOOH** | Self-Harm | 20 | N |
| **EDSSS** | Alcohol Intoxication | 192 | Y |
|  | Overdoses | 214 | Y |
| **GPIH** | Prescriptions | 818 | Y |
| **NASS** | Overdoses | 530 | N |

**Notes:** NHS111 – National Health Service 111 telephone service; GPOOH – General Practitioner Out-of-Hours; EDSSS – Emergency Department Syndromic Surveillance System; Surveillance; GPIH – General Practitioner In-Hours; NASS – National Ambulance Syndromic Surveillance System; N/A – Not Available

**Table S5.** Percentage differences between actual and counterfactual utilisation for MH indicators.

| **System** | **Period \ Indicator** | **PRL1** | **L1** | **PL1** | **L2** | **PL2** |
| --- | --- | --- | --- | --- | --- | --- |
| **EDSSS** | Overdoses | -3.3  (-7.2, 0.4) | -22.2  (-27.5, -17.4) | 3.0  (-0.4, 6.1) | -4.6  (-7.8, -1.7) | 7.9  (4.4, 11.1) |
|  | Alcohol intoxication | -9.5  (-15.4, -4.2) | -35.8  (-44.0, -28.4) | -1.0  (-5.8, 3.4) | -19.0  (-24.1, -14.4) | 2.7  (-2.5, 7.3) |
| **NASS** | Overdoses | 2.3  (-1.1, 5.6) | -27.0  (-32.1, -22.3) | -9.5  (-13.0, -6.3) | -13.3  (-16.4, -10.3) | 3.1  (-0.3, 6.3) |
| **GPIH** | Prescriptions | -12.2  (-17.7, -7.3) | -26.3  (-33.1, -20.2) | -13.0  (-17.9, -8.5) | -3.9  (-7.7, -0.4) | -0.1  (-4.8, 4.2) |
| **NHS 111** | Sleep Difficulties | -16.0  (-27.7, -6.3) | 19.6  (10.2, 27.3) | 20.4  (13.1, 26.5) | 34.5  (29.4, 38.8) | 26.3  (18.6, 32.7) |
| **GPOOH** | Self-harm | -25.7  (-41.7, -12.9) | -15.9  (-31.8, -3.3) | -8.3  (-19.9, 1.3) | 37.5  (31.2, 42.8) | 2.7  (-8.9, 12.0) |

**Notes:** Values are % difference with 95% confidence intervals. Orange or blue font items have 95% CI entirely above or below zero. Orange font is higher utilisation than counterfactual; blue font highlights lower than expected presentations. **PRL1:** Pre-lockdown 1: February 25 to March 22, 2020; **L1:** Lockdown 1: March 23 to May 31, 2020; **PL1:** Post Lockdown 1: June 1 to November 4, 2020; **L2:** Lockdown 2: November 5, 2020 to March 7, 2021; **PL2:** Post-Lockdown 2: March 8 to July 7, 2021. NHS111 – National Health Service 111 telephone service; GPIH – General Practitioner In-Hours; GPOOH; General Practitioner Out-of-Hours; EDSSS – Emergency Department Syndromic Surveillance System

**Figure S1.** GP in Hours prescriptions for mental health conditions and calls to NHS111 about sleep difficulties.


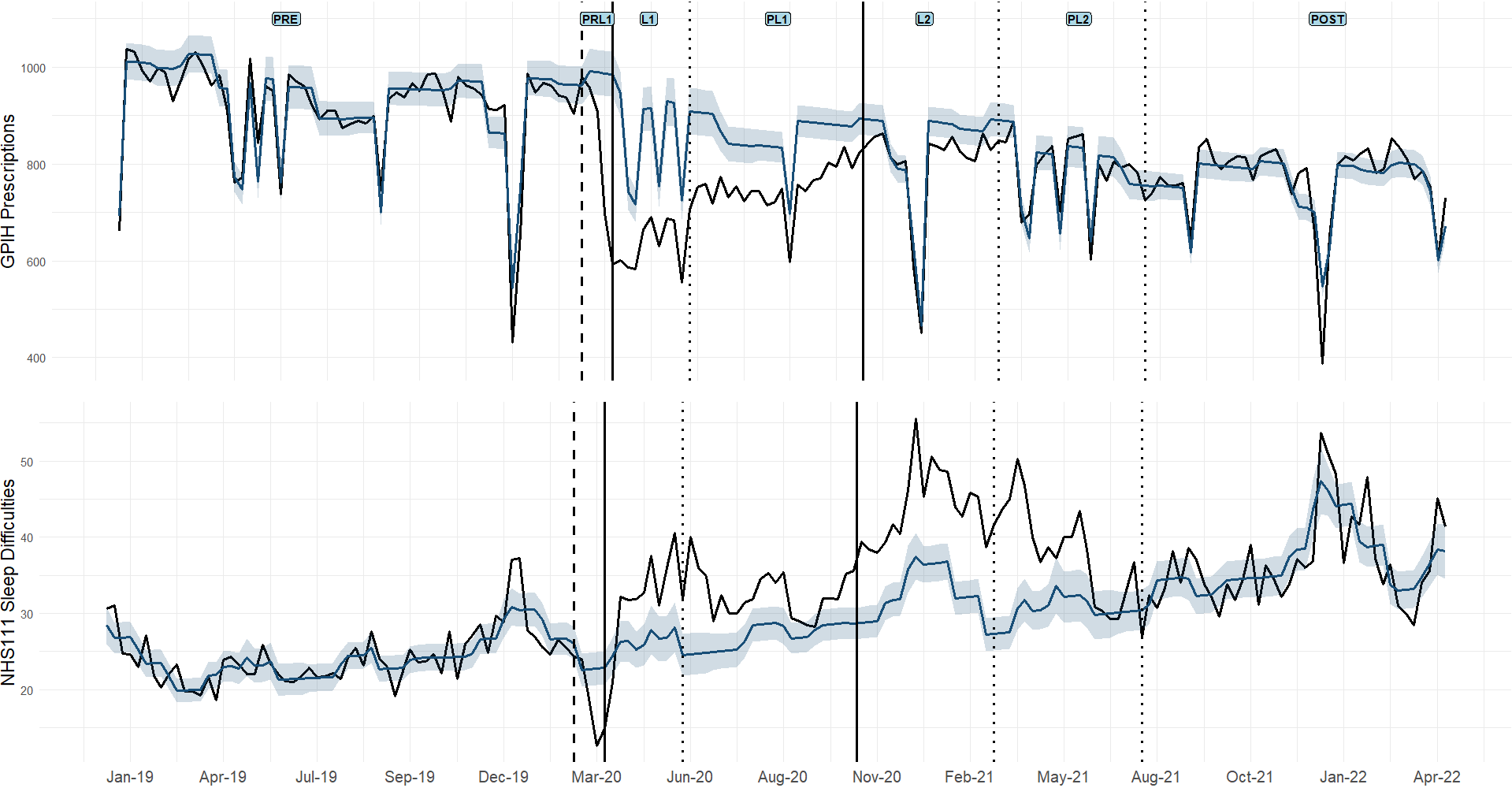


Notes: Vertical lines represent starts of different time periods.

**Figure S2.** Mental health indicators : emergency department attendances for alcohol intoxication or alcohol overdose; ambulance attendances for overdoses and GP out of Hours calls for self-harm events


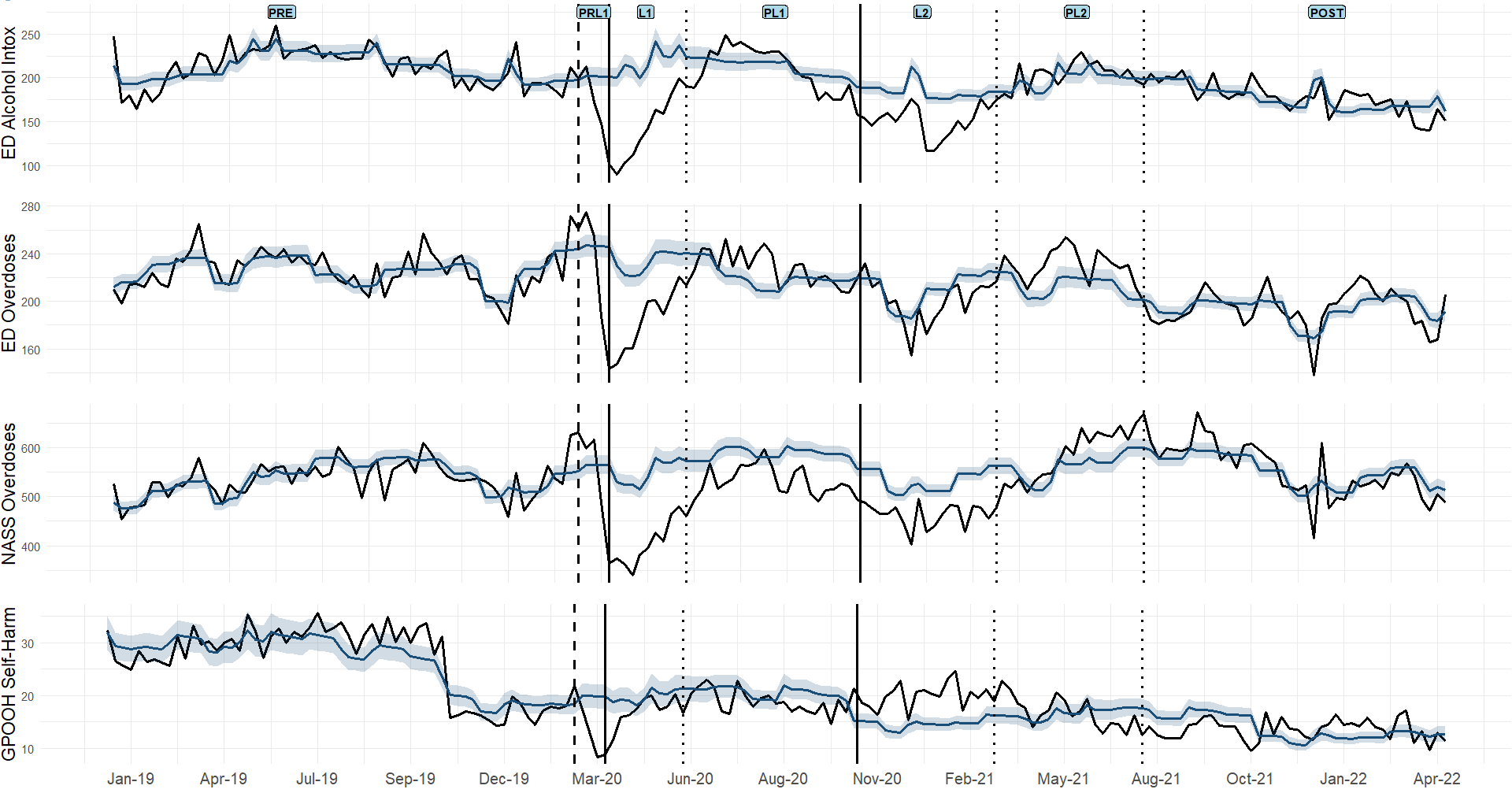


Notes: Vertical lines represent starts of different time periods

**Figure S3: Forest Plot of GP-In-Hours Mental Health Indicators Stratified by Age Group.** Percent change compared with counterfactual estimates is displayed on the x-axis, with a spaced vertical line during each period representing a 0% change. Each individual indicator and their respective surveillance system is reported on the Y-axis.


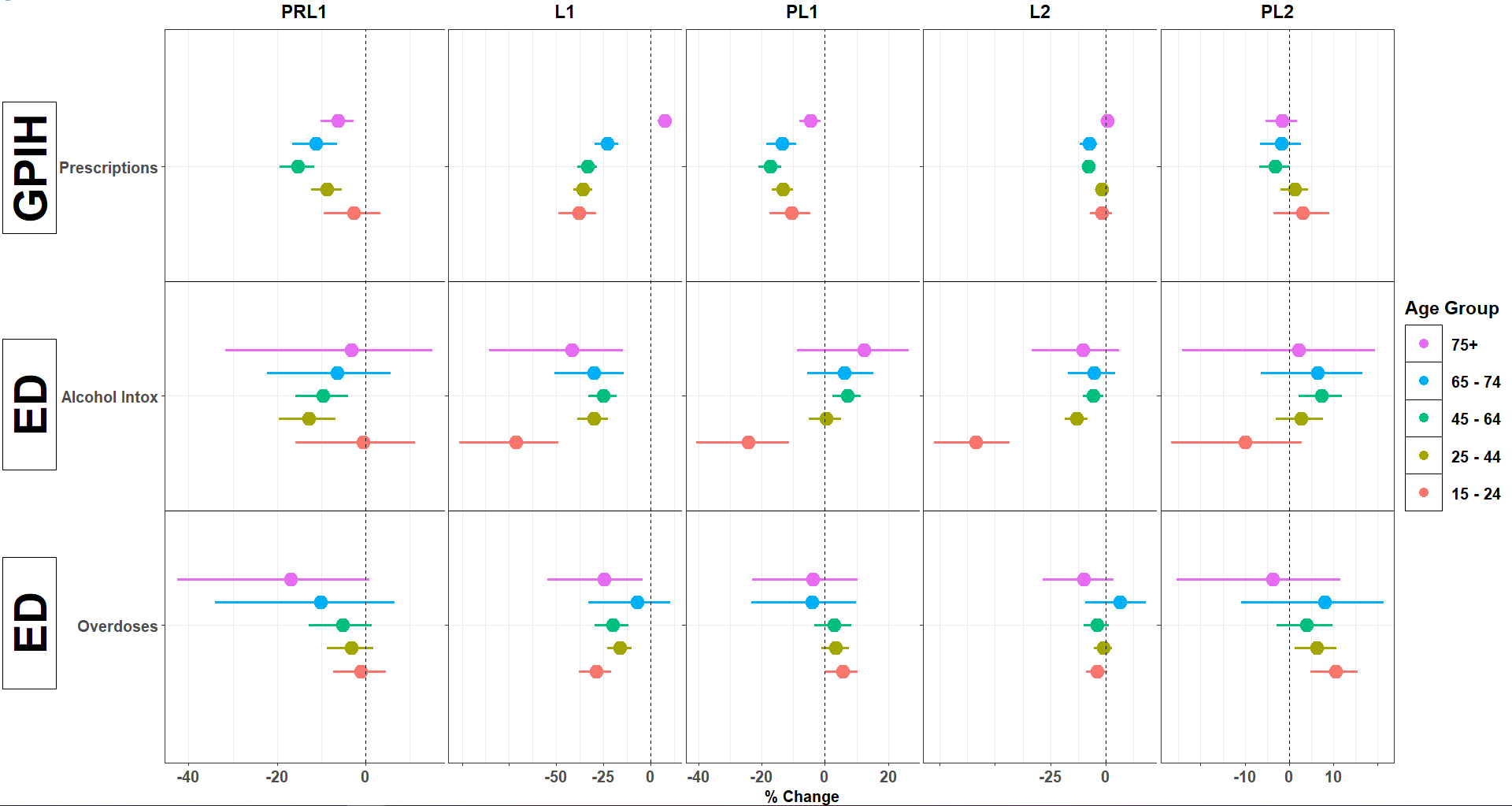


**Figure S4: Forest Plot of GP-In-Hours Mental Health Indicators Stratified by sex.** Percent change compared with counterfactual estimates is displayed on the x-axis, with a spaced vertical line during each period representing a 0% change. Each individual indicator and their respective surveillance system is reported on the Y-axis.


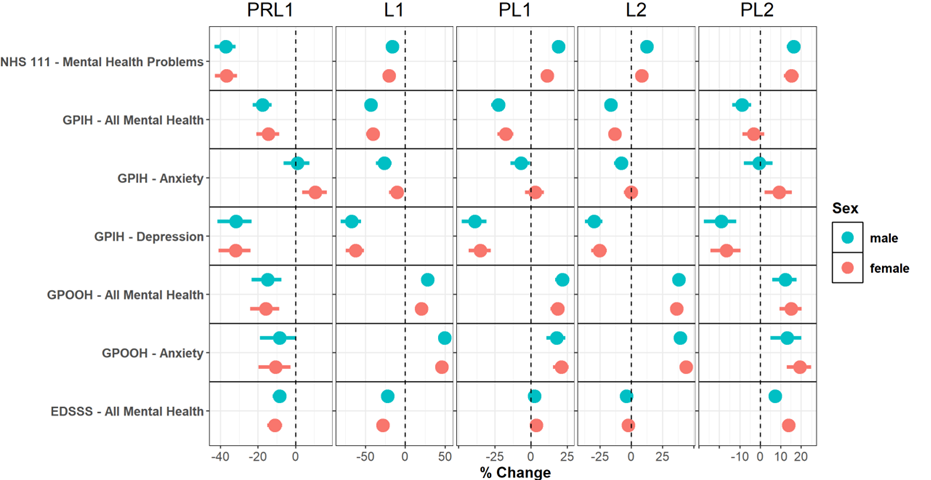


**Figure S5: Forest Plot of GP-In-Hours Mental Health Indicators Stratified by Gender.** Percent change compared with counterfactual estimates is displayed on the x-axis, with a spaced vertical line during each period representing a 0% change. Each individual indicator and their respective surveillance system is reported on the Y-axis.


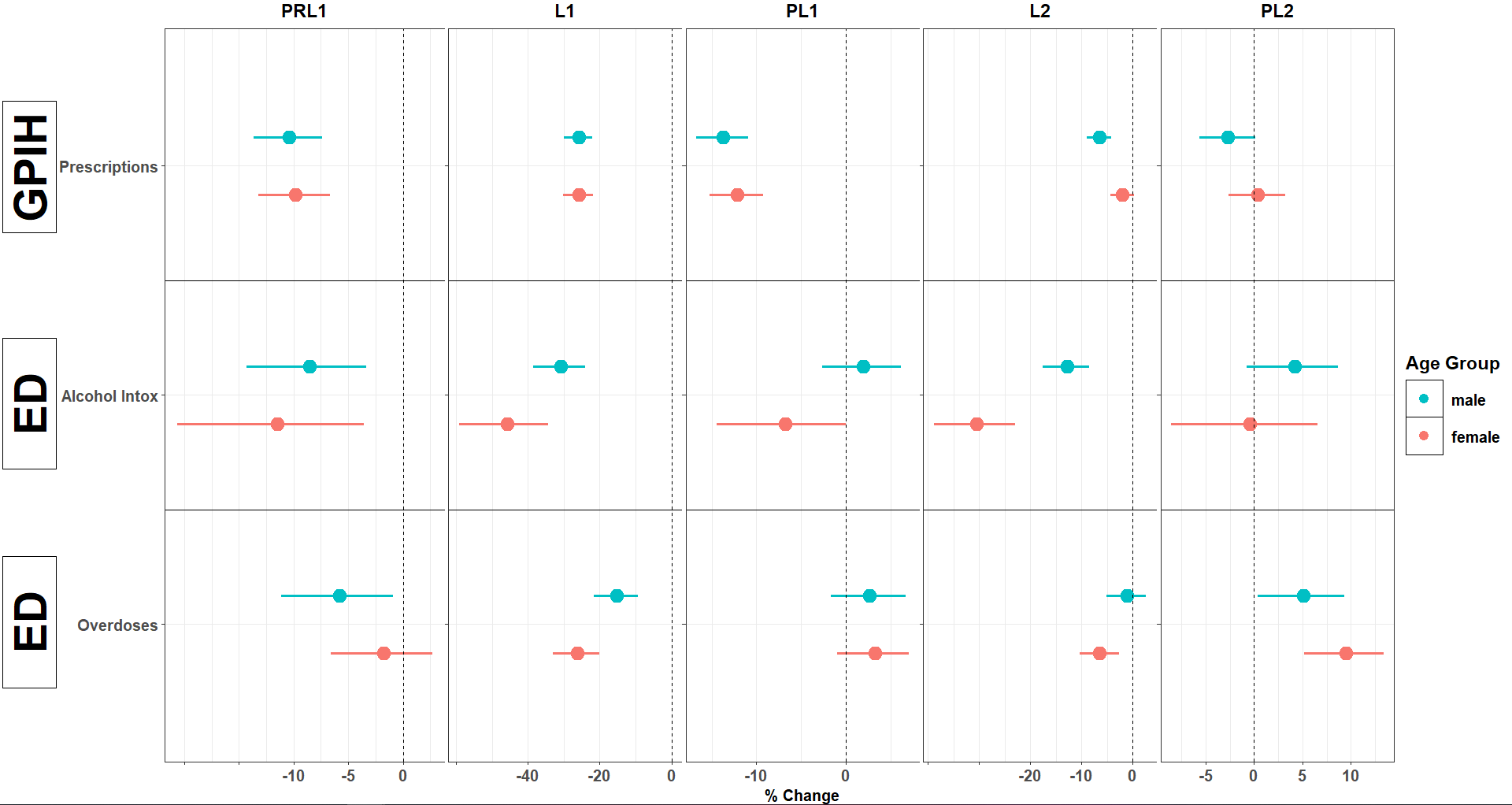

Supplement: Supplementary file 1 — Supplementary Material 1 [file 12913_2026_14362_MOESM1_ESM.docx]
